# Case Report: Targeted treatment by fluoxetine/norfluoxetine of a *KCNC2* variant causing developmental and epileptic encephalopathy

**DOI:** 10.3389/fphar.2024.1528541

**Published:** 2025-01-15

**Authors:** Ping Li, Alice Butler, Yu Zhou, Karl L. Magleby, Christina A. Gurnett, Lawrence Salkoff

**Affiliations:** ^1^ Department of Neuroscience, Washington University in St. Louis, St. Louis, MO, United States; ^2^ Department of Anesthesiology, Washington University in St. Louis, St. Louis, MO, United States; ^3^ Department of Physiology and Biophysics, University of Miami Miller School of Medicine, Miami, FL, United States; ^4^ Department of Neurology, Division of Pediatric and Developmental Neurology at Washington University in St. Louis, MO, St. Louis, United States

**Keywords:** Prozac, fluoxetine, norfluoxetine 4-fluoro-7-nitro-2,1,3-benzoxadiazole, potassium channel, epilepsy, autism, *KCNC2* electrophysiology, Kv3.2

## Abstract

The Kv3.2 subfamily of voltage activated potassium channels encoded by the *KCNC2* gene is abundantly expressed in neurons that fire trains of fast action potentials that are a major source of cortical inhibition. Gain-of-function (GOF) *de novo* pathogenic variants in *KCNC1* and *KCNC2*, encoding Kv3.1 and Kv3.2 respectively, cause several types of epilepsy including developmental and epileptic encephalopathy (DEE). Fluoxetine (Prozac) is a known inhibitor of the Kv3.1 current and was reported to improve seizure control in a single patient with a *KCNC1* GOF variant. Here, we describe fluoxetine treatment of two siblings with a *de novo KCNC2* V473A variant associated with DEE, which resulted in improved seizure control, ability to wean antiepileptic medications, and improved development. The *KCNC2* V437A variant showed GOF activity as demonstrated by HEK293 cells expressing variant subunits activating at more hyperpolarized potentials than WT channels. Fluoxetine reduced currents equally for both Kv3.2 WT and Kv3.2-V473A variant channels, with an IC_50_ of ∼12 µM. Further analysis of this repurposed drug showed that norfluoxetine, a long-lasting metabolite of fluoxetine which is produced in the liver and accumulates in the brain, was more effective than fluoxetine itself in selectively inhibiting the dominant pathogenic channel activity of the pathogenic allele. Norfluoxetine showed 7-fold greater selectivity in inhibiting Kv3.2 variant currents (IC_50_ of ∼0.4 µM) compared to WT currents (IC_50_ of ∼2.9 µM). Combined with a previous report of improved outcomes for a *KCNC1* variant, our results suggest that fluoxetine or its metabolite, norfluoxetine, may be beneficial for patients with GOF variants in *KCNC2* and other neuronal potassium channels.

## Introduction

The utility of genetic testing for epilepsy diagnoses is becoming increasingly realized, particularly as it improves pharmacological treatments of seizures and selection of medications based on precision therapeutics. This has been especially useful in treatment of patients with genetic variants in ion channel genes, such as *SCN1A* (Dravet syndrome) and other sodium channelopathies, including *SCN2A* whose seizures are known to worsen or improve with sodium channel blockers (reviewed in (Anwar et al., 2019)).

Not unexpectedly, pathogenic variants in genes encoding potassium channels that shape and control electrical signaling in the brain are increasingly being uncovered as causes of severe epilepsies. In particular, many developmental and epileptic encephalopathies (DEEs) are associated with *de novo* variants in neuronally expressed genes encoding voltage-dependent K^+^ channels (Vetri et al., 2024). Two highly similar paralogues of voltage-activated potassium channel genes, *KCNC1* and *KCNC2*, which encode Kv3.1 and Kv3.2 potassium channels, are expressed in neurons that fire at high frequency, such as GABAergic interneurons in neocortex, hippocampus, caudate nucleus and thalamocortical projections (Gutman et al., 2005; Rudy and McBain, 2001; Kolodin, 2008). GABAergic inhibition produced by this rapid firing is an important regulator of excitability in neuronal networks and its alteration by GOF mutations in these genes can lead to intractable epilepsy as well as other neurological disease (Kaar et al., 2024). The presence of Kv3.1 and Kv3.2 K^+^ channels in inhibitory circuits suggest that GOF variants may be particularly prone to causing serious pathology when the rapid firing pattern is disrupted (Kaar et al., 2024).

Pathogenic variants of *KCNC1* are associated with severe neurological phenotypes, including myoclonic epilepsy, ataxia, intellectual disability without epilepsy, and developmental and epileptic encephalopathy (Ambrosino et al., 2023; Clatot et al., 2023). While many *KCNC1* variants have loss-of-function (LOF) properties, three out of 11 variants showed gain-of-function (GOF) properties when studied *in vitro*. In a heterologous channel expression system, fluoxetine inhibited both the Kv3.1 WT and Kv3.1 V425M GOF channels. Based on this result, fluoxetine was successfully used to treat a DEE patient harboring a *de novo* missense *KCNC1* GOF variant (V425M) resulting in sustained improvement in seizure control, as well as balance, gross motor skills, and oculomotor coordination (Ambrosino et al., 2023).

Patients with *KCNC2* variants have similar types of epilepsy with respect to age of onset and severity (Li et al., 2022; Schwarz et al., 2022). The majority of previously described *KCNC2* variants are *de novo* pathogenic variants, whereas the others have been considered as possible modifier variants. *De novo KCNC2* variants often result in DEE, with age at seizure onset ranging from the first month of life to 1 year. Ataxia, hypotonia, autism, hyperactivity, early-onset absence epilepsy, and DEE have been reported, with functional analysis showing GOF in more severely affected individuals with DEE (Li et al., 2022; Schwarz et al., 2022). Although valproate was reported to result in seizure freedom for some *KCNC2* patients, many patients with *KCNC2* variants had drug resistant epilepsy (Schwarz et al., 2022).

Here we describe the treatment of two siblings with a *KCNC2* GOF variant (Mehinovic et al., 2022) with fluoxetine, a drug previously used to successfully treat a child with a *KCNC1* GOF missense variant (Ambrosino et al., 2023). Treatment of siblings carrying the *KCNC2* V473A GOF variant led to significant clinical amelioration, with reduction in seizure frequency and improved function. To explore the action of fluoxetine, as well as its major metabolite norfluoxetine (Eli-Lilly and Company, 2006), we undertook heterologous expression of WT and variant KV3.2 channels to demonstrate that fluoxetine reduced currents for both Kv3.2 WT and Kv3.2-V473A variant channels. Norfluoxetine was more effective than fluoxetine, indicating a more selective action of norfluoxetine on V473A variant channels than WT. The reduction of currents by fluoxetine, and its major metabolite norfluoxetine, may provide a mechanism for the observed therapeutic effects. Fluoxetine and norfluoxetine are known open channel blockers of Kv3.1channels (Choi et al., 2001; Sung et al., 2008) and may act in a similar manner for the closely related Kv3.2 channels.

Note: Methods have been included as a supplemental section.

## Results

### Case description

The probands are sisters, age 27 and 25, who both presented with epilepsy around 6 weeks of life. Seizures were described as eye fluttering during the transition between wakefulness and sleep. EEG showed generalized and multifocal epileptiform abnormalities. Seizure types consist mostly of atypical absence seizures with activity arrest and stereotypical ictal behavior including eye flutter, drooling, and head drop, and infrequent myoclonic seizures. Both were diagnosed with DEE, hypotonic cerebral palsy, autism, and intellectual disability, and ultimately failed multiple treatments for epilepsy, including phenobarbital, ACTH, prednisolone, carbamazepine, rufinamide, clobazam, felbamate, and Epidiolex. Their seizures partially responded to the ketogenic diet and valproic acid. Brain MRI of sibling #1 was normal except a cavum septum pellucidum, and sibling #2 had bilateral ventriculomegaly, prominent in the posterior horns and trigones of the lateral ventricles with slight thinning of the posterior body of the corpus callosum. There was no family history of epilepsy. Diagnostic genetic testing, which was not available until they were nearly adults, demonstrated *de novo KCNC2* missense variant resulting in a V473A substitution in both siblings as previously identified (Mehinovic et al., 2022). Developmentally, sibling #1 walked independently at age 6, uses gestures, vocalizations, and augmentative devices for communication, and follows 2-3 step commands. Sibling #2 walked independently at age 2 and communicates with short sentences and follows multi-step commands.

### Clinical response to fluoxetine

**FIGURE 1:**
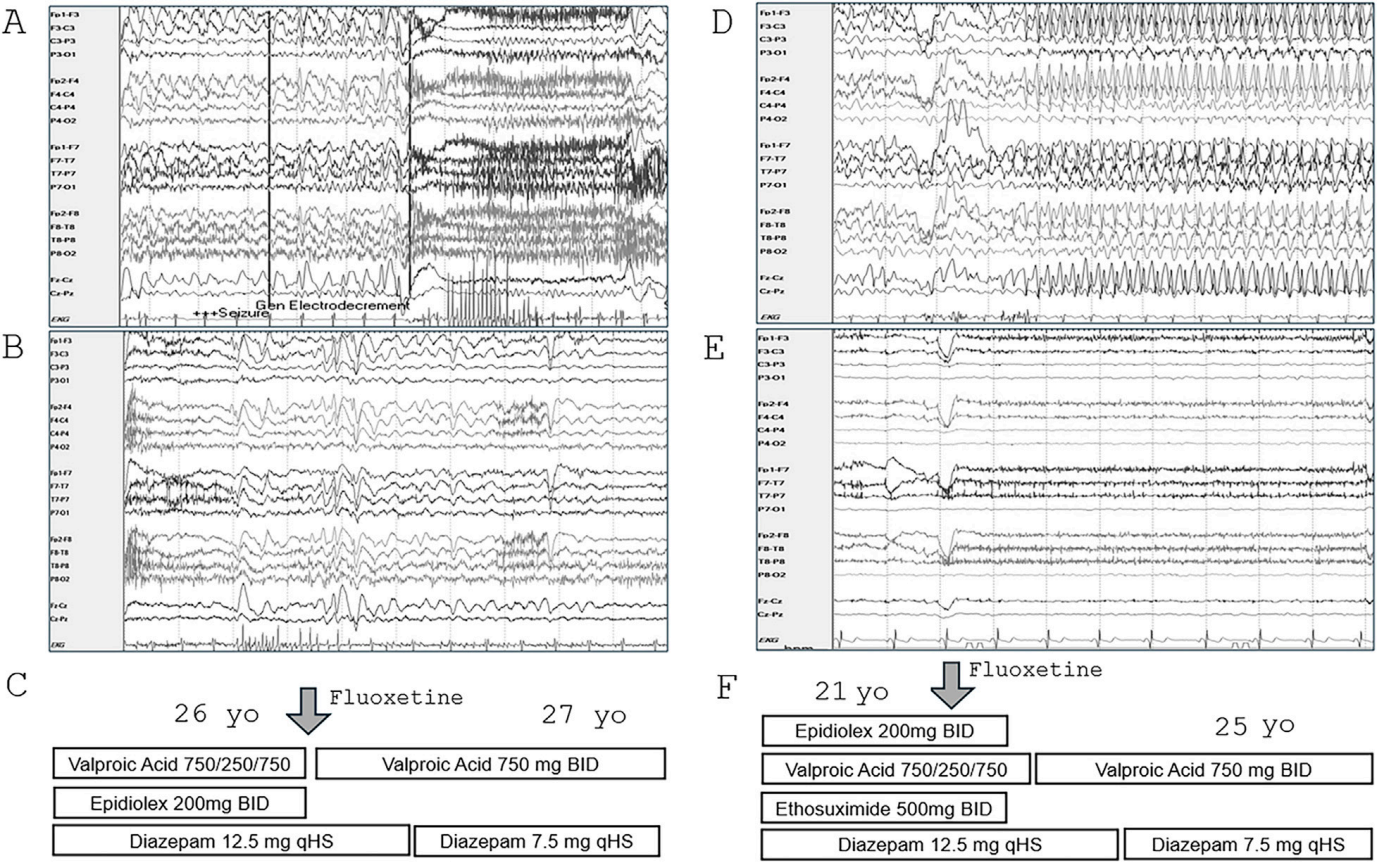
Improvement in EEG and antiepileptic medication response in siblings treated with fluoxetine. **(A)** Selected EEG from overnight recording in sibling #1 (age 25) showing nearly continuous 3–4 Hz frontally predominant generalized discharges and electrodecrement associated with atypical absence seizure. **(B)** Routine EEG in sibling #1 (age 27), 2 months after starting fluoxetine, showed bifrontal slowing and frequent bursts of generalized discharges but no seizures. **(C)** Anti-epileptic medications were reduced following fluoxetine. **(D)** Selected EEG during overnight recording in sibling #2 (age 20) showing nearly continuous 3–4 Hz frontally predominant generalized discharges and atypical absence seizures. **(E)** Four years after starting fluoxetine, overnight EEG (age 25) showed slow background activity with no epileptiform abnormalities or seizures. **(F)** Anti-epileptic medications were reduced following fluoxetine.

Treatment with fluoxetine was initiated in sibling #1 at age 26, Her atypical absence seizures occurred 3–5 times/day with incontinence and post-ictal period in the month prior to starting fluoxetine 20 mg/day and were reduced to 0–1 times/day with no incontinence or post-ictal period during the following month. The seizure frequency was anecdotal and not formally quantified by either caregiver or provider. By the family’s report, she also demonstrated marked improvement in stamina that eliminated the need for an adaptive stroller, verbalizations, mood, and new skill development, including opening doors and initiating independent ambulation. Overnight EEG prior to fluoxetine, at age 25, showed nearly continuous 3–4 Hz frontally predominant generalized discharges, with recording of 2 atypical absence seizures associated with electrodecrement (Figure 1A). A routine EEG (age 27) was improved and showed no seizures (Figure 1B). After fluoxetine was initiated, she was able to discontinue Epidiolex and reduce Depakote and Diazepam (Figure 1C).

Treatment with fluoxetine 20 mg was initiated in sibling #2 at age 21 for impulsivity and irritability. Seizure frequency was reduced from 1-2 seizures/day in the month prior to 1-2 seizures/week during the following year. The seizure frequency was anecdotal and not formally quantified. By the family’s report, she had marked improvement in expressive communication, alertness, and reduction in irritability, outbursts, and aggression. Prior to fluoxetine, at age 20, overnight EEG showed nearly continuous 3–4 Hz frontally predominant generalized discharges and 6 brief electroclinical atypical absence seizures (Figure 1D). Overnight EEG at age 25 showed slow background activity with no epileptiform abnormalities or seizures (Figure 1E). Ethosuximide, Epidiolex, and risperdone were discontinued, and diazepam and valproic acid dosages were reduced (Figure 1F). Higher doses of fluoxetine were tried but were not tolerated due to irritability.

### *KCNC2* V473A variant is located in S6 domain

**FIGURE 2:**
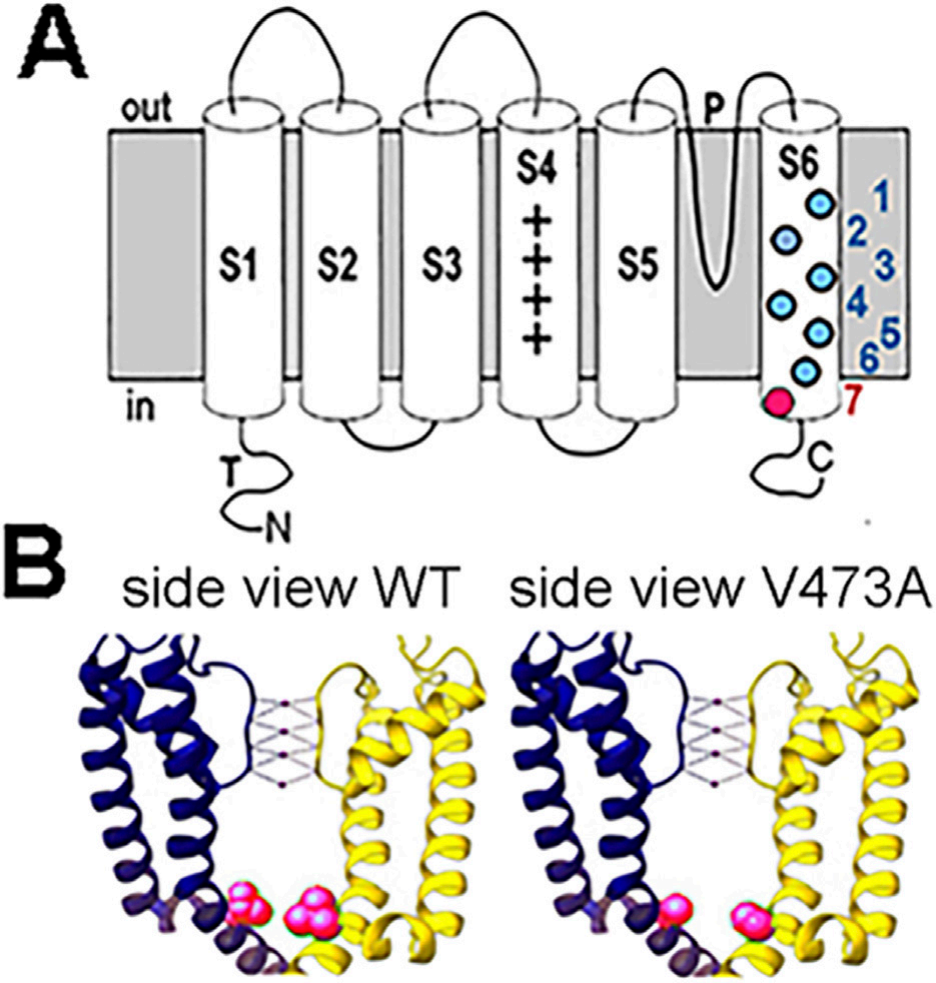
Diagram of a Kv3 potassium channel subunit and the location of the V473A variant. **(A)** Kv3 subunit with 6 transmembrane domains. P indicates the pore domain governing the ion selectivity of the channel. The colored filled circles in S6 represent selected dominant gain-of-function variants in Kv3.1 and Kv3.2. The KV3.2 V473A variant is located near the cytoplasmic opening of the channel pore (red). Kv3.1 variants: 1) V425M, 3) M430I, 4) V432M, 5) V434L (Ambrosino et al., 2023); KV3.2 variants: 2) I465V, 6) V471L (Li et al., 2022; Schwarz et al., 2022), 7) V473A (Schwarz et al., 2022) (which is the topic of this study). **(B)** Position of WT V473 and V473A variant on S6 in the Kv3.2 channel structure. The WT V473 (red) is located near the cytoplasmic mouth of the channel and may be important in stabilizing the closed structure (left image). The variant V473A is shown in the right image (red). Structures of human Kv3.2 were based on the cryo-EM structure of the highly similar paralog, Kv3.1, where the S6 amino acid sequence is identical for Kv3.1 and Kv3.2. The residue position V436 in the Kv3.1 sequence is equivalent to residue V473 in Kv3.2 [P48547 *KCNC1* compared to Q96PR1 *KCNC2* - UniProt]. The images of human Kv3.1 structure were prepared in UCSF Chimera (Pettersen et al., 2004). The V436A (V473A) variation in the Kv3.1 (Kv3.2) structure was generated from the WT Kv3.1 cryo-EM structure (PDB: 7PHH) by replacing V436 with Alanine using the Swapaa function in UCSF Chimera (right image).

The *KCNC2 V473A de novo* variant is located within the sixth membrane spanning domain (S6) that supports the contour of the internal vestibule and is allosterically linked to the voltage sensor, regulating the voltage-dependent opening and closing of the channel (Figure 2A). The channel’s main activation gate, which closes off the potassium conductance pathway, is located at the helical bundle crossing region of the S6 segments on the cytoplasmic face of the channel (Chi et al., 2022; Kim and Nimigean, 2016; Mukherjee et al., 2022). Notably, the proline-valine-proline (PVP) motif, also called the S6 gating hinge, is located between 468 and 470. The V473A mutation is located very close to the bundle crossing (Figure 2B) which is involved in the mechanical flexibility of the pore and regulation of the activation energy necessary to open the channel. Kv3.2, encoded by *KCNC2*, is a highly similar paralog of Kv3.1 (*KCNC1*) and has an identical S6 amino acid sequence. In fact, there are only 2/174 amino acid differences in sequence inclusive of S4 through S6 so the structure is assumed to be similar. The residue position V473 in the Kv3.2 sequence is equivalent to residue V436 in Kv3.1 because of a length difference in an amino acid segment near the amino terminal that shifts the residues by 37. While not all GOF variants in Kv3.1 and Kv3.2 are located in this region, the clustering of GOF mutations in S6 (Figure 2A) may indicate that GOF mutations in this region shift the equilibrium among the open and closed states to favor the open states.

### The pathogenic Kv3.2-V473A variant activates at more hyperpolarized voltages compared to WT Kv3.2 currents, indicating a GOF variant

**FIGURE 3:**
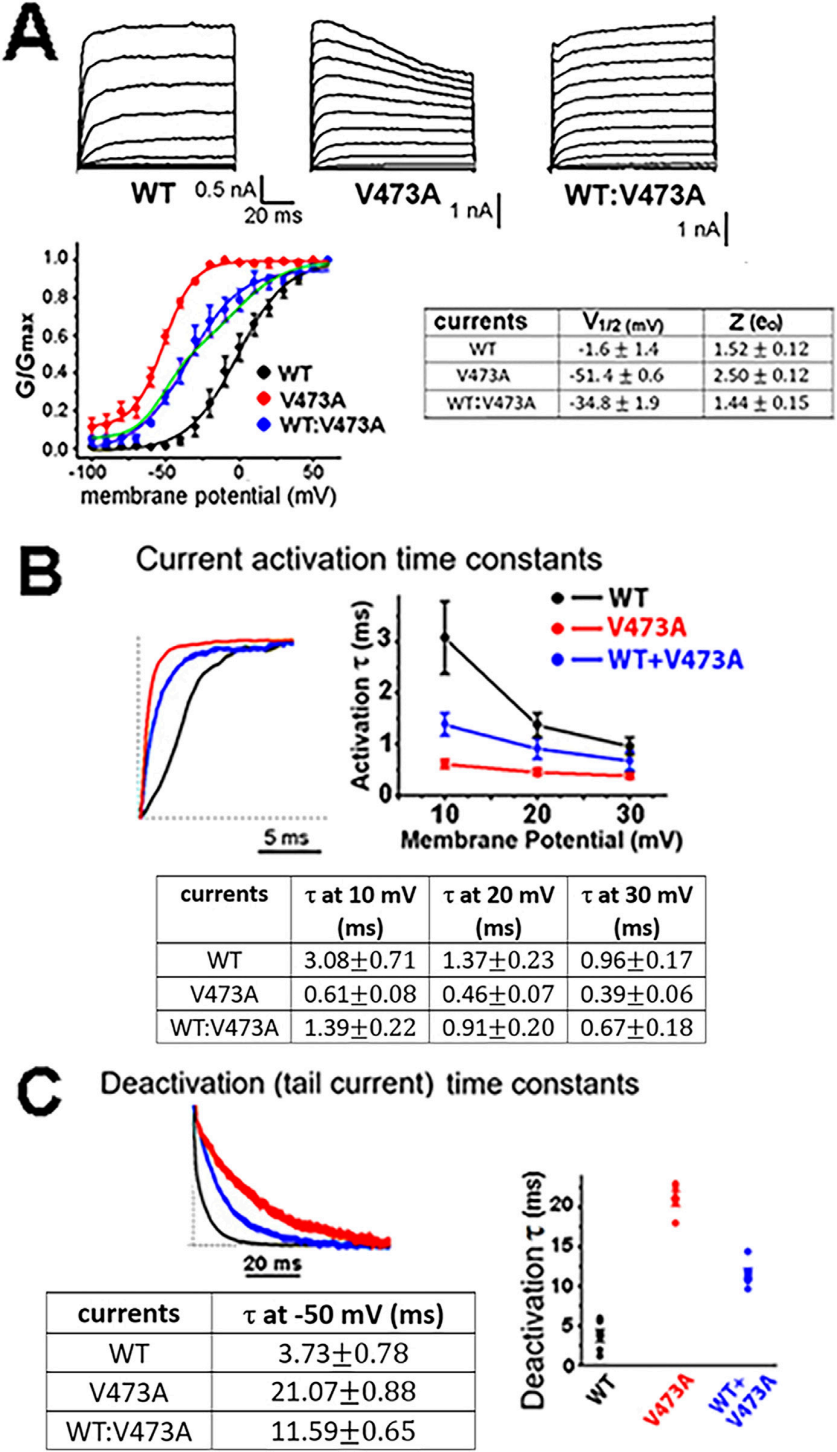
*KCNC2* K^+^ currents expressed by human Kv3.2 WT channels, pathogenic variant V473A channels, and a 1 to 1 co-expression of WT and V473A subunits indicate that V473A subunits cause a GOF variant. **(A)** HEK293 cells were transiently transfected with vectors expressing subunit cDNAs as indicated. Membrane voltage was held at a holding potential of −80 mV and subjected to a series of increasing 10 mV depolarizing step pulses. The WT:V473A currents shown on the far right may resemble those in the heterozygous patients and are likely to be expressed from a group of channels having different stoichiometry’s made up of different numbers of WT and V473A variant subunits, as was the case for a BK channel GOF variant (Labro et al., 2015). The currents differ greatly in their voltage range of activation, as shown in the conductance/voltage (G/V) plots (n = 5, *p* < 0.001). The current expressed by the channels composed of the variant subunit alone (red) is shifted leftward −50 mV to a more negative voltage range compared to WT currents. The variant current is also more voltage sensitive as indicated by the steeper G/V plot and a higher Z (gating charge) value as shown on the right. The 1 to 1 co-expression of WT and mutant Kv3.2 subunits produces a current with a G/V curve that is shifted to negative voltages (blue symbols) intermediate between WT (black symbols) and variant (red symbols). As was the case for the WT and variant currents, the intermediate WT:V473A currents were also well fit by a single Boltzmann function but with a shallower slope, as would be expected if channels with a range of V1/2 values contribute to the intermediate currents (Geng et al., 2023). Most of the channels from co-expression are likely to be hybrid channels (Geng et al., 2023; Supplementary Figure S1). Note that the G/V current for WT:V473A co-expression is poorly fit if it is assumed that WT and V473A subunits only self-assemble without the formation of hybrid channels, as shown by the green line which is the sum of two separate Boltzmans, one for WT channels and one for variant channels. **(B)** The *KCNC2* variant V473A causes Kv3.2 currents to activate more rapidly than WT channels. Activation currents for the indicated channel types for a step from −80 mV to +40 mV (left) with plots and a table of the activation time constants for voltage steps from −80 mV to the indicated three voltages. **(C)** V473A variant channels remain open longer, as indicated by the slower deactivation time constants. Plotted deactivation currents (left) for the three channel types were for voltage steps from +40 mV to −50 mV. Faster activation and slower deactivation for channels with variant V473A subunits occurred at all voltages examined (n = 5, *p* < 0.001). Channels arising from mimicking a heterozygous mutation of WT and V473A variants had properties intermediate between WT and V473A channels.

To investigate the functional effect of the siblings *KCNC2* V473A variant, we used heterologous channel expression in mammalian HEK293 cells together with whole cell patch clamp recording and found that currents from human WT Kv3.2 channels activate with an inflection delay (Figure 3A) and remain activated for the duration of the examined depolarizations, as described previously (Kaczmarek and Zhang, 2017; Lien and Jonas, 2003). The half-activation voltage (V_1/2_) of the WT Kv3.2 currents was −1.6 ± 1.4 mV, which is more positive than that of most other voltage gated channels (Lien and Jonas, 2003; Pak et al., 1991a; Pak et al., 1991b). A V_1/2_ near 0 mV for the WT Kv3.2 channels would permit the rapid unimpeded rise of the action potential (Lien and Jonas, 2003). In comparison, currents associated with the pathogenic Kv3.2-V473A variant differed markedly from the WT Kv3.2 currents. Currents for the Kv3.2-V473A variant rose more rapidly and the voltage for half-activation of current was left-shifted by more than 50 mV along the voltage axis to more negative potentials compared to WT Kv3.2 currents.

Because all reported *KCNC2* pathogenic variants, including the Kv3.2-V473A variant described here, occur in the heterozygous state, random assembly of WT and variant subunits would be expected to result in five types of channels with different stoichiometries in an individual patient. Three of the five types would be hybrid channels comprised of both WT and variant subunits in various combinations, with lessor numbers of WT and homomeric mutant channels (Supplementary Figure S1; MacKinnon, 1991; Blaine and Ribera, 1998). To mimic a heterozygous variant, HEK cells were transfected 1:1 with equal amounts of WT Kv3.2 and variant Kv3.2-V473A expression vectors. As might be expected for these 1:1 “heterozygous” transfections, the V1/2 for the G/V curve (Figure 3A, blue line in plotted data) falls between that of the WT Kv3.2 current (black line) and the Kv3.2-V473A currents (red line), likely mimicking the currents present in cells of the heterozygous sisters. These currents were well described by a single Boltzmann function (blue line). We showed previously that currents generated by the random assembly of WT and variant subunits of an analogous GOF BK channel mutation produced a mix of five channel stoichiometries where the G/V curves were well approximated by a single Boltzmann function (Geng et al., 2023). In contrast, the G/V curve of the hybrid currents was less well described by a single Boltzmann function if we assumed that the 1:1 currents did not form hybrid currents and arose from two separate populations of channels, half homomeric WT channels and half homomeric variant channels (Figure 3A plotted data, green curve).

Channels formed exclusively from four mutant subunits express a current with an initial component of inactivavation. Significantly, such inactivation is not seen in the 1 to 1 expression of the mutant and WT subunits. Thus, such inactivation is not likely to be a significant component of the potassium current in the heterozygous patients which may have a mix of channels with stoichiometries resembling those represented in Supplementary Figure S1. It is possible that only a small percent of channels in the patients (∼6%) consisting of four mutant subunits have such inactivation.

In addition to their different voltage range of activation, the Kv3.2-V473A variant currents activated 5-fold more rapidly than WT Kv3.2 currents (Figure 3B) following depolarizing voltage steps and then deactivated 5.6-fold more slowly than WT Kv3.2 currents following negative voltage steps (Figure 3C). The negative shift in the voltage range of activation, 5-fold faster activation and 5.6-fold slower deactivation, would all increase the time that Kv3.2-V473A variant currents were active, indicating that Kv3.2-V473A is a gain of function variant. These changes are consistent with a shift in the gating properties of the variant channels favoring the open state.

### Fluoxetine and norfluoxetine both block Kv3.2 channels; norfluoxetine is most effective and targets the variant

**FIGURE 4:**
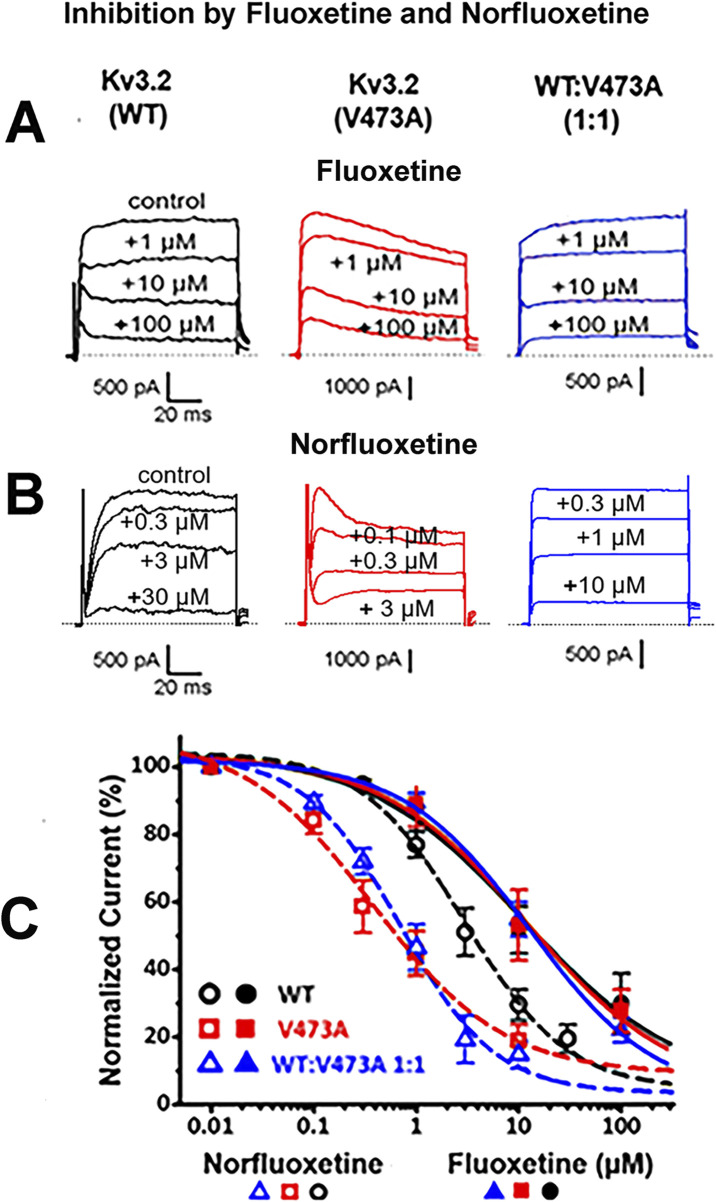
Fluoxetine and norfluoxetine inhibit currents from WT Kv3.2 channels, pathogenic variant V473A channels, and from channels expressed following a 1:1 transfection of WT and V473A subunits. **(A)**. Representative current traces showing a dose dependent block by fluoxetine. **(B)**. Representative current traces showing a dose dependent block by norfluoxetine. Voltage steps were from −80 mV to +40 mV with current measurements at +30 mV. Drug concentrations are indicated. Currents were recorded from whole HEK cells following transfections for the indicated subunits. **(C)** Dose response curves showing block of all three Kv3.2 K^+^ channel types by fluoxetine and norfluoxetine, as indicated. Fluoxetine blocked the different channel types similarly: For WT, the IC_50_ was 11.4 ± 5.84 µM with a Hill coefficient of 0.56 ± 0.13 (n = 6); for V473A, 12.2 ± 5.93 µM with a Hill coefficient of 0.61 ± 0.11 (n = 5); for WT:V473A (1:1) the IC_50_ was 12.8 ± 2.91 µM with the Hill coefficient of 0.71 ± 0.06 (n = 5) (*p* = 0.968). Norfluoxetine blocked all three Kv3.2 K^+^ channels more effectively than fluoxetine, especially the K^+^ currents carried by Kv3.2 V473A channels and the heteromeric 1 to 1 ratio of WT and V473A. For WT (n = 7) the IC_50_ was 2.91 ± 0.63 µM with the Hill coefficient of 0.86 ± 0.08; for V473A (n = 6) the IC_50_ was 0.38 ± 0.15 µM with the Hill coefficient of 0.68 ± 0.09; for WT:V473A (1:1) (n = 6) the IC_50_ was 0.69 ± 0.14 µM with the Hill coefficient of 0.94 ± 0.08 (*p* < 0.001).

Previous work demonstrated that fluoxetine resulted in clinical improvement in a patient with a *de novo KCNC1* GOF missense variant (V425M) (Ambrosino et al., 2023), and inhibited both WT and mutant Kv3.1 channels (as well as hybrid channels), with a slight bias toward inhibition of the variant current (Ambrosino et al., 2023). Because *KCNC1* and *KCNC2*, the paralogous genes encoding the Kv3.1 and Kv3.2 Shaw family potassium channels, share large regions of identical structure and closely overlapping anatomical distributions in the brain, we decided to investigate whether there might be a similar channel blocking effect of fluoxetine on Kv3.2 currents, which might be clinically useful for individuals with *KCNC2* GOF variants. As shown in Figures 4A, C, fluoxetine similarly blocked the K^+^ currents carried by homomeric WT Kv3.2 channels, homomeric V473A channels, and channels assembled from WT and V473A subunits to mimic a heterozygous mutation, where most of the channels could be heteromeric (Geng et al., 2023). Thus, the effect of fluoxetine on Kv3.2 channels is similar to that on their close paralogue Kv3.1.

Norfluoxetine, a metabolite of fluoxetine (Eli-Lilly and Company, 2006), has been shown to be considerably more effective in blocking Kv3.1 channels than fluoxetine itself (Choi et al., 2001). Fluoxetine is converted into norfluoxetine in the liver by removal of a methyl group and only a small percentage of both agents (<5%) is estimated to be eliminated by the kidney (Eli-Lilly and Company, 2006). In patch clamp experiments using the heterologous HEK293 cell expression system, we found that norfluoxetine was indeed a more potent blocker of Kv3.2 currents than fluoxetine, by a factor of about 3.6 for WT currents (Figure 4). Furthermore, norfluoxetine discriminates between WT and variant Kv3.2 currents; the inhibition by norfluoxetine on variant currents is approximately seven times greater than on WT currents (Figure 4).

### Physiological role of Kv3.2 channels and the potential impact of a GOF Kv3.2 mutant on the excitability of fast spiking neurons

Kv3.2 and also Kv3.1 channels activate (open) at voltages that are far more positive than the action potential threshold (4). Indeed, these channels have a specialized mechanism of conductance called a “resurgent” current in which the channels, upon depolarization, rapidly transit into a specialized closed state and then rapidly open on the downward (repolarizing) phase of the action potential (Labro et al., 2015). The channels then rapidly deactivate to minimize the refractory period of the action potential. This resurgent mechanism thus facilitates rapid repolarization while allowing the rapid regeneration of the next action potential. Rather than producing a delayed resurgent Kv3.2 current to repolarize the action potential, the mutant V473A channel produces a voltage dependent potassium current which activates at a more negative membrane voltage (Figure 3A) and activates more rapidly than WT Kv3.2 channels (Figure 3B). Thus, its more rapid activation at negative voltages supersedes the action of the resurgent current (Labro et al., 2015), which normally does not greatly contribute to action potential repolarization until the downward, repolarizing phase of the action potential. The left shift in activation, more rapid activation and decreased rate of deactivation of the pathological GOF variant Kv3.2 (V473A) (Figure 3) would all negatively alter the properties of fast spiking neurons by removing specializations in Kv3.2 that support fast spiking. To visualize the impact of Kv3.2V473A on the neuronal excitability of fast spiking neurons, we simulated the action potential response of a PV interneuron model embedded with WT or mutated Kv3.2 channels to current injection. The result shows that the pathogenic phenotype could both reduce the frequency of firing in a train of action potentials and potentially eliminate repetitive firing (Supplementary Figure S2; Murakoshi and Trimmer, 1999; Santi et al., 2006; Liu and Bean, 2014; Olah et al., 2022). Notably, the complete elimination of the Kv3.2 current in the simulation is less damaging to repetitive firing than including the pathological currents contributed by the GOF Kv3.2 (V473A) variant, which is consistent with the effects of a genetic deletion of the Kv3.2 current in mouse models (Lau et al., 2000).

## Discussion

Fluoxetine is a known open channel blocker of Kv3.1 channels (Ambrosino et al., 2023), and here we describe similarly potent inhibitory effects on Kv3.2 channels. Fluoxetine and norfluoxetine both appear to be open channel blockers with norfluoxetine being the physically smaller of the two because of its loss of a methyl group during metabolism in the liver. Conceivably, loss of the methyl group could improve access or binding to the channel. While fluoxetine blocked the WT Kv3.2 currents similarly to its block of V473A variant currents, we found a significant increase in potency for norfluoxetine blocking V473A variant currents vs. WT Kv3.2 currents. Further investigation may determine whether this higher potency to block variant V473A channels is related to a greater open time of the variant due to faster opening, slower closing, and negative shifted activation. During steady state administration of fluoxetine, norfluoxetine rises to concentrations in the serum approximately as high as fluoxetine (Henry et al., 2005; Sagahon-Azua et al., 2021). In brain, both may rise to concentrations higher than in serum because of the lipophilic environment of the brain (Henry et al., 2005; Renshaw et al., 1992).

In addition to norfluoxetine blocking both Kv3.2 WT and V473A channels more effectively than fluoxetine, norfluoxetine is also a more potent selective serotonin reuptake inhibitor (SSRI) than fluoxetine (Eli-Lilly and Company, 2006; Wong et al., 1993) but has not been marketed by pharma in this role because its effects on a cardiac potassium channel are associated with long-QT syndrome (Magyar et al., 2004). Voltage clamp experiments on cardiac tissue also revealed a concentration-dependent suppression of norfluoxetine on both L-type Ca^2+^ current, I(Ca) (EC_50_ = 1.13 ± 0.08 µM) and transient outward K^+^ current, I (to) (EC_50_ = 1.19 ± 0.17 µM). However, its effectiveness at blocking the Kv3.2 V473A variant current at low concentrations might render it effective at inhibiting the variant current without cardiac side effects. Significantly, a postmortem study of tissues of patients treated with fluoxetine showed that its metabolite norfluoxetine was present in the brain at concentrations equal or higher than that of fluoxetine (Lewis et al., 2007). Fluoxetine is also recognized as a blocker of the persistent component of sodium conductance which is known to have an anti-seizure effect (Igelstrom and Heyward, 2012). Thus, the effects of fluoxetine on both Kv3.2 currents and persistent sodium currents, along with its action as a SSRI, may additively combine to confer the ameliorative benefit of fluoxetine. It should be noted, however, that both excitatory and inhibitory cortical neurons have sodium channel types known to produce persistent sodium current (such as NaV1.6 and NaV1.1) (Katz et al., 2018) and it is difficult to predict what the net effect would be in this circumstance.

There is accumulating data regarding the utility of drug repurposing for treating patients with potassium channelopathies. While valproic acid was previously reported to be beneficial in some patients with *KCNC2* variants, it was only partially effective in the siblings reported here who had significant benefit when fluoxetine was added. It was previously shown that repurposing fluoxetine improved seizure control and neurodevelopment in a DEE patient with a GOF variant in *KCNC1* (Ambrosino et al., 2023), and we found that repurposing fluoxetine was effective in treating the two patients described here with *KCNC2* GOF variant (V473A), indicating the utility of repurposing drugs.

Controlled clinical trials of fluoxetine for patients with potassium channelopathies are needed to assess effects on seizures and neurodevelopment with emphasis on GOF variants as examined here. Furthermore, the higher potency of norfluoxetine for Kv3.2 V473A variant currents suggests that chemical modifications may be able to provide further selectivity for channel subtype and/or variant and reduce toxicity. However, the advantages of repurposing fluoxetine, one of the most commonly prescribed psychotropic drugs are compelling, and support an urgent need for translational research on its use in patients with DEE-associated GOF potassium channelopathies.

## Conclusion

Next-generation sequencing techniques have greatly advanced our understanding of the genetic causes and mechanisms behind developmental and epileptic encephalopathies (DEEs). Identifying specific genetic defects in patients can help tailor treatments based on the particular functional differences resulting from these mutations. We showed that a novel pathogenic variant (V473A) in the *KCNC2* gene, encoding Kv3.2 voltage-gated potassium channel, caused GOF functional properties when expressed in a heterologous channel expression system and that fluoxetine treatment, most likely in conjunction with its metabolite norfluoxetine, led to seizure suppression and behavioral improvement of two siblings. The relationship between fluoxetine and norfluoxetine with regard to their relative contributions requires further clarification and large-scale studies are needed to establish whether treatment with these, or other related compounds, might provide similar clinical benefits in patients with other pathogenic GOF variants in *KCNC2* or related potassium channels.

## Data Availability

The raw data supporting the conclusions of this article will be made available by the authors, without undue reservation.
