# Supplementary material for: Case Report: Targeted treatment by fluoxetine/norfluoxetine of a KCNC2 variant causing developmental and epileptic encephalopathy
Source: Front Pharmacol. 2025 Jan 15;15:1528541. doi: 10.3389/fphar.2024.1528541 (PMC11774886; doi:10.3389/fphar.2024.1528541)
Supplement: Supplementary file 1 [file Supplementaryfile1.docx]

**
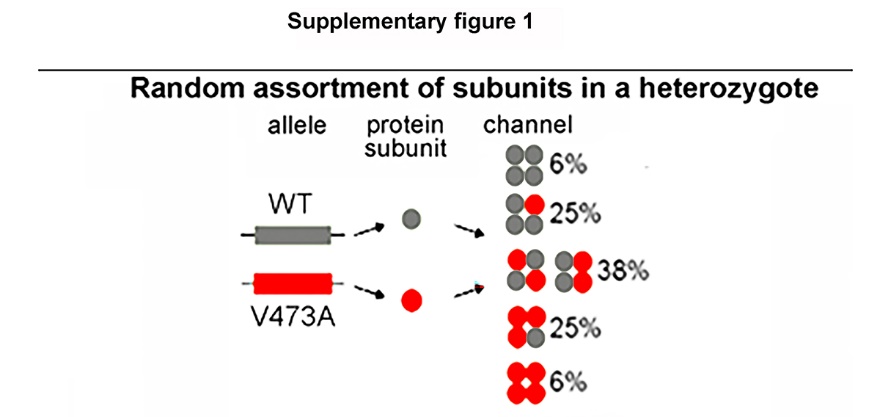
**

**Legend: Supplementary Figure S1. Predicted subunit composition of channels for a heterozygous genotype assuming random assembly of WT and variant subunits.** Random assembly predicts 87.5% hybrid (heteromeric) channels, with only 6.25% homomeric WT (gray) and 6.25% homomeric mutant (red) channels. This hypothetical distribution for random assembly was found to be consistent for a BK channel variant G375R heterozygous with WT by applying single channel recording and analysis [22]. Note that the vast majority (93.75%) of channels from random assembly could have one or more variant subunits, potentially contributing to the pathological phenotype. The use of the binomial equation for calculating the percentages is from [34] as modified by [35].

**
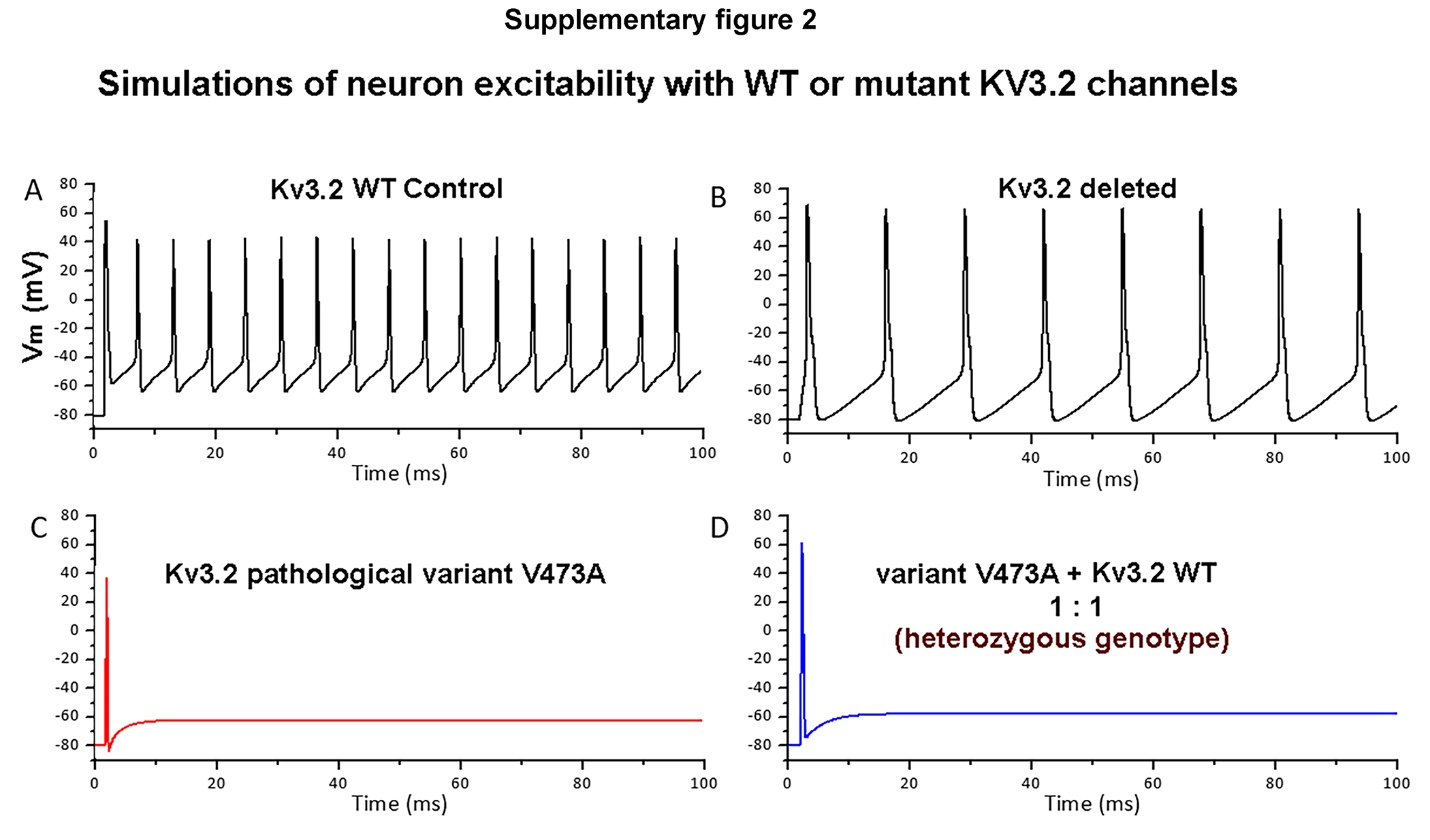
**

**Legend: Supplementary Figure S2. Simulation of a fast spiking neuron excitability with WT and variant Kv3.2 channels.** The channel densities in the cells are identical for all 4 scenarios except for when Kv3.2 channels are not present (B). In all other panels, two voltage-dependent channels are present, Kv3.2 (WT or variant as stipulated) and Kv2.1 (Kv2.1 channels are known to be present in the majority of mammalian neurons [36]). **(A)** WT Kv3.2 channels result in fast repetitive firing. **(B)** Deleting the WT Kv 3.2 channels slows the firing rate. **(C)** Replacing the WT Kv3.2 channels with variant V473A Kv3.2 channels reduces repetitive firing to only an initial active response. **(D)** Replacing WT Kv3.2 channels with a 1 to 1 mixture of WT and V473A channels, to approximate the conditions of a heterozygous patient (Fig. 3A) reduces repetitive firing to an initial active response. More details are given in Supplementary Table 1.

**Supplementary Table 1.**

**The gating parameters of Kv3.2 and Kv2 used in NEURON simulation**

|  | Vh1 | actsteep | acttauavg | acttaupeak | acctauoffset | acttauwdt |
| --- | --- | --- | --- | --- | --- | --- |
| Kv3.2WT | -01.6 | 16.8 | 2.88 | 15.2 | 38.6 | 10.6 |
| Kv3.2V473A | -51.4 | 10.1 | 0.05 | 26.7 | 41.5 | 14.4 |
| WT+V473A | -34.8 | 18 | 0.8 | 13.2 | 41.3 | 16.23 |
| Kv2 | -07.2 | 14.6 | 5 | 67.8 | 22.6 | 11.2 |

**Legend for Supplementary Table 1**. The gating parameters of Kv2 and Kv3.2 channels (Table 1) were determined from published data [38] or experimentally determined from this lab (Kv3.2: G-V and tau-V relationships).

Kv2 or Kv3 is described by a Hodgkin–Huxley model in which the time course of Kv open probability m(t) is defined by following set of equations:

$$\begin{matrix} \frac{dm(t)}{dt}=\frac{m_{inf}\left( V \right)-m(t)}{m_{\tau}(V)} \\ m_{inf}\left( V \right)=1-\frac{1}{1+\exp((V+Vh1)/actsteep)} \\ m_{\tau}\left( V \right)=acttauavg+acttaupeak*\exp(-0.5*((V+acttauoffset)/{acttauwdt}^{2})) \end{matrix}$$

In above equations, m*_inf_*(V) and m_t_(V) are the steady-state open probability and activation/deactivation time constant at voltage V, respectively. The gating parameters (Vh1, actsteep, acttauavg, acttaupeak, acttauoffset and acttauwdt) of Kv2 and Kv3.2 channels are determined from published (Kv2, Liu PW and Bean, BP, JNS, 2014) or experimentally determined (Kv3.2) G-V and tau-V relationships.

The biophysical features of our model such as the volume, sodium channel type and density, resistance and capacity are configured to mimic that of a PV-positive GABAergic neuron. Upon request we can provide the source codes as the ultimate detail of the simulation,

**SUPPLEMENTARY DATA: MATERIALS AND METHODS**

**Subject recruitment.** The patients were identified through a sequencing study described previously [11]. Participation in this study was conducted with informed consent of the research participants under the Washington University School of Medicine Institutional Review Board. EEG were recorded using the international 10-20 standard electrode placement.

**HEK293 cell culture.** As described previously [39], human embryonic kidney HEK293 cells (ATCC CRL-1573; ATCC) were cultured in Gibco Dulbecco’s modified Eagle’s medium (DMEM) with 10% fetal bovine serum, 100 units/ml penicillin, and 100 μg/ml streptomycin (Thermo Fisher Scientific) and incubated at 37°C with 5% CO_2_. These cells were grown and passaged twice a week in T25 flasks (MidSci).

**Expression of Kv3.2 channels in HEK293 cells.** HEK293 cells were plated at a density of ∼400,000 cells per 40-mm petri dish (#93040; TPP catalog) a day before transfection. For transfection, plasmid DNA containing cDNA encoding for KV3.2 WT and/or KV3.2 V473A (mutant) subunits was added to cell layers that were 70–90% confluent using Lipofectamine 2000 transfection reagent (catalog #11668-027; Thermo Fisher Scientific) following manufacturer’s instructions. 2 μg cDNA of WT, mutant, or a mix of WT and mutant (1:1 ratio) was transfected per 40 mm dish. As a marker for transfection, cells were cotransfected with pmaxGFP, a CMV plasmid expressing green fluorescent protein (Amaxa Biosystems) at 0.2 μg per dish. After transfection, the cells were incubated at 37°C with 5% CO_2_ for 2–4 d until recording.

**Whole-cell recording from HEK293 cells.** Details of recording methods for similar experiments have been described recently [22]. In brief, whole-cell recordings were obtained from HEK293 cells using an Axopatch 200B amplifier (Molecular Devices, Sunnyvale, CA). Data were acquired with the Clampex program from the pClamp software package (Molecular Devices, San Jose, CA). Recordings were filtered at 5 kHz with the amplifier internal filter and digitized at 50 kHz using a Digidata 1550B digitizer (Molecular Devices). Recording pipettes were pulled from borosilicate glass with tip resistances of 3–8 MΩ after filling with pipette solution containing (in mM): 140 KMES, 1 EGTA, 10 HEPES, pH 7.4 with KOH. Bath solutions contained (in mM) 135 NaMES, 5 KMES, 2 MgCl_2_, and 10 HEPES, pH 7.4 with NaOH. Experiments were performed at room temperature (~22°C). Fluoxetine and norfluoxetine were prepared daily from a 10 mM stock solution in DMSO. The steady-state currents evoked at +30 mV were normalized to the values obtained in the absence of compounds to generate dose–response curves.

**Data Analysis.** Data were analyzed with pClamp 10.6 (Molecular Devices). The G-V relationship of Kv3.2 channels was determined from tail currents measured 150 ms after repolarization to −50 mV. G-V curves were fit by a Boltzmann function:

$$G(V)=\frac{G_{\max}}{1+\exp(-z(V-V_{h})/kT)}$$

in which *G*_max_ is maximal conductance, *z* is apparent voltage-dependence in units of elementary charge, *V*_h_ is the voltage of half-maximal activation, and *k* and *T* have their usual physical meanings.

The Fluoxetine/Norfluoxetine inhibition dose-response relationships were fit by the Hill equation:

$$R=\frac{1}{1+(C/IC_{50})^{n}}+I_{off}$$

in which *R* is fractional unblock, *C* is Fluoxetine or Norfluoxetine concentration, IC_50_ is half inhibition concentration, *n* is Hill coefficient, and I_off_ is an offset item for leak current. Data were analyzed using OriginPro 7.5 (OriginLab Corporation) or programs developed in this laboratory. Error bars in the figures represent SEMs and error estimates in the text are SD. Each data point is averaged from at least 5 measurements. Curve fitting results are reported as the fitted values with standard error, which was estimated by OriginPro according to the Error Propagation formula. Significance was determined with one-way ANOVA unless otherwise indicated.
